# Ethnophytotherapeutical research in the high Molise region (Central-Southern Italy)

**DOI:** 10.1186/1746-4269-4-7

**Published:** 2008-03-11

**Authors:** Paolo Maria Guarrera, Fernando Lucchese, Simone Medori

**Affiliations:** 1 Museo Nazionale Arti e Tradizioni Popolari, Piazza Marconi 8-10, 00144 Rome, Italy; 2 Dipartimento di Biologia, Università di Roma Tre, Viale Marconi 446, 00146 Rome, Italy

## Abstract

**Background:**

In the years 2003–2005 research was carried out concerning ethno-medicine in the high Molise (central- southern Italy), a region that has been the object of very little investigation from the ethnobotanical point of view. Upper Molise is a continuation of the mountain profiles of the Abruzzi Appenines: a series of hills, steep slopes and deep fluvial valleys making communications difficult. Primordial traditions (e.g. harvest feasts) are typical of the region.

**Methods:**

Field data were collected through open interviews in the field. These were conducted on both an individual and group level, fresh plants gathered from surrounding areas being displayed. In other cases, individual interviews were conducted by accompanying the person involved to the places where they perform their activities (for example, in the woods with woodcutters, kitchen gardens and fields with housewives, pastures with shepherds, etc.). In total 54 individuals were interviewed.

**Results:**

Data of 70 taxa belonging to 39 families were gathered. Among the species, 64 are used in human therapy, 5 as insect repellents, 11 in veterinary medicine, 1 to keep eggs and cheeses and 4 for magic purposes. The most important findings in ethno-medicine relate to the lichen *Lobaria pulmonaria *(L.) Hoffm. (wounds) and to some vascular plant species: *Asplenium trichomanes *L. and *Ceterach officinarum *Willd. (to regularize menstruation), *Cyclamen hederifolium *(chilblains), *Centaurium erythraea *Rafn. and *Pulmonaria apennina *Cristof. & Puppi (bruises), while in the ethno-veterinary field, we have *Valeriana officinalis *L. (wounds sustained by mules). Also worthy of note, given the isolation of the area, is the number of plants used to protect foodstuffs from parasites, among which *Allium sativum *L. and *Capsicum frutescens *L.

**Conclusion:**

The research revealed a deep-rooted and widespread habit of husbanding the family's resources. Whilst isolation and snowfalls contributed to the widespread knowledge of means of conserving foodstuffs, they also led to the use of products easily available within each home. The values of E.I. (ethnobotanicity index) for the upper Molise region are considered amongst the highest in Italian areas. Nevertheless, like the values for other areas of Italy, they are lower than those of many Spanish areas, perhaps (and not only) because of the more rapid cultural erosion experienced in Italy.

## Background

The primary aim of this research activity, conducted between February 2003 and February 2005, is to provide an initial picture of the ethnobotany in the Molise region, and the only Italian area not previously studied from this viewpoint, with the exception of a brief paper by Guarrera [1]. The region does not have an autonomous regional administrative identity since it was incorporated into Abruzzo until 1963, a factor which has not helped stimulate research into its territory as an independent entity. A second reason it that, given its climatic and morphological characteristics, the area presents certain interesting elements of local isolation, which is probably one of the main reasons why there is a differentiation in the typical usages in the region . The existing ethnographic literature concerning the Molise is scarce and out of date [2-4], with the exception of a few works [5,6] and mainly relates to ritual aspects and beliefs.

**Figure 1:**
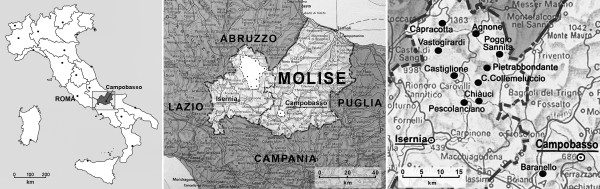


The area under study (Fig. 1), covering 377,76 square km, lies in the province of Isernia, in Upper Molise, in the council areas of Pietrabbondante, Chiauci, Poggio Sannita, Castiglione di Carovilli, Vastogirardi, Agnone, Pescolanciano, Pescopennataro and Capracotta, and in the province of Campobasso, in the council area of Baranello (total inhabitants: 15235) [7,8]. The central point of the research is in the council area of Pietrabbondante, 1027 m a.s.l. and bordering on the MAB riserve of Collemeluccio. In terms of landscape and geo-morphology, Upper Molise is a continuation of the mountain profiles of the Abruzzi Appenines: a series of hills, steep slopes and deep fluvial valleys making communications difficult [9]. Rocky spurs jut out from the stony slopes favouring the establishment of settlements such as Pietrabbondante, for example. Yellowish sandy arenaceous facies and limestone seams at high altitudes are typical of the area.

The council areas studied present a wide variety of environments, ranging from 700 to 1400 m a.s.l. Man's exploitation of the terrain so as to create space for cereal crops has had the effect of fragmenting the terrain which is now a heterogeneous mosaic variously shaped parcels of land. The stony soil is not very suitable for cultivation; for this reason many people have emigrated. In Roman times the inhabitants of this region were shepherds and very strong warriors. The Molise region has records of this civilisation in the ruins of some towns (Bovianum Vetus, Saepinum etc). The studied area was inhabited by the ancient Sanniti Caraceni (or Carecini) and Pentri people [10,11]. The dialects of the upper Molise region (particularly the dialect of Agnone) are similar to those of neighbouring Abruzzo, above all (but not only) in terms of phonetics. Instead the dialects of middle Molise (e.g. that of Baranello) are more closely linked to the dialects of Campania [12-16]. Primordial traditions are typical of the region, e.g. harvest feasts and characteristic torches made with *Abies alba *L. at Christmas [17,18].

The climate is continental given the mountain range and currents of cold air from the north and north-east during the winter, and of warm, humid air in the summer. The high altitude results in frequent and abundant snowfalls [9]. The area includes an enormous variety of vegetation (beech and fir woods, mixed groves of oaks and pastures). The MAB reserves of Collemeluccio and Montedimezzo fall into the *Polysticho-Fagetum aceretosum pseudoplatani *varying to *Acer lobelii *Ten., the *Aquifolio-Fagetum fraxinetosum excelsioris*, and *Aquifolio-Fagetum abietetosum albae*, also including *Quercus cerris *L. [9,19]. The flora of the entire Molise region consists of 2422 taxa [9], while in the areas studied in this research about n. 800 taxa grow (F. Lucchese, personal communication).

## Methods

Data was gathered by means of open interviews in the field, once the aims of the research had been outlined and permission obtained for publication of the data collected. The authors take full responsibility for the declaration that the people interviewed are aware that the information they have provided will be published.

A questionnaire was used in interviews, variations being introduced during the interviews themselves. These were conducted on both an individual and group level, fresh plants gathered from surrounding areas being displayed. This proved extremely efficient from the quantitative point of view, with large quantities of data being obtained, albeit sometimes circumstantial. In other cases, individual interviews were conducted by accompanying the person involved to the places where they perform their activities (for example, in the woods with woodcutters, kitchen gardens and fields with housewives, pastures with shepherds, etc.). This approach was aimed at understanding the specific knowledge of the individual in relation to his or her particular profession and environment and proved to be an extremely valid method in qualitative terms. The data thus collected often related to a smaller number of plants but furnished a greater quantity of specific information. In recording information of diseases, care was taken not to "translate" this into medical terms, but to refer exactly whatever was reported, so as to avoid the information being in any way falsified.

The plants were identified with the use of the Flora d' Italia by Pignatti [20] and its nomenclature was updated by Conti et al. [21]. Voucher herbarium specimens were kept in the Herbarium of the Faculty of Biological Sciences at the University Roma Tre (acronym URT).

A total of 54 individuals were interviewed (roughly 65% women, 35% men), most of whom were aged between 70 and 90 (max. 92, min. 40, mainly between 70 and 80, mean about 74) and mainly woodcutters, farmers and housewives. For reasons of privacy, it was possible to obtain specific complete personal data relating to only 15 informants. The data collected (see Additional file 1, Tables 1, 2) include the scientific and dialect names of the plants, the parts used, preparation, local uses, frequency of mention, eventual current usage, period in which the plant is gathered and kind of habitat in which the plant grows.

**Table 1:** Veterinary uses of plants in the high Molise region

**Family, scientific name, voucher specimen**	**Vernacular names**	**Parts used**	**Preparation**	**Local uses**	**Number of informants and locality**	**Present use**	**Period of gathe-ring**	**Habitat**
**Caprifoliaceae**								
*Sambucus nigra *L. MEA14	Sammùche	Leaves	Decoction (e.u.)	It was applied to wounds in mules	1(CP)	No	Al	
**Compositae**								
*Sonchus oleraceus *L. LU4	Cacignilli (CA)	Aerial part	Fried in oil and applied as poultice (use against venom)	Insect bites (horse-flies etc.) of livestock (horses, cows) or bites of other animals	3(CA)	No	Al	Unc, wa, ru
**Gramineae**								
*Triticum aestivum *L. LU9	Grano	Stems	Burnt straw applied with oil in a poultice	In veterinary medicine on wounds of mules	1(CP)	No	Su	
**Liliaceae**								
*Allium sativum *L. LU15	Aglio	Bulb	A ball of lard with garlic inside	To eliminate worms in dogs (as food)	1 (PE)	Yes		
**Malvaceae**								
*Malva sylvestris *L. subsp. *sylvestris *MEA4	Maula	Aerial part	Decoction for washes	Cows udders (wounds, reddening)	1(AG)	No	Al	
**Oleaceae**								
*Olea europaea *L. LU19	Olivo	Oil	Mixed with soot	Open wounds in livestock	6(PI) 2(CA)	Yes rare		
		Oil	Mix with oil, sugar, chalk and 'green clay, this last present near spring	Broken cattle horns (cementing mixture)	1(PI)	No		
		Oil	Warmed in frying pan ('olio ferrato')	Applied to small wounds of livestock	6(PI)	No		
**Ranunculaceae**								
*Clematis vitalba *L. MEB6	Vitalba	Stem	Wrapped 7 times around the neck of sheep and left a week	Veterinary/ritual use for mad sheep ('scapocciate')	1(PE)	-	Al	Wo, he
**Ulmaceae**								
*Ulmus minor *Miller LU27	Olmo	Bark, root	Decoction	Cicatrizing agent for wounds	1(PE)	No	Al	Daw
**Urticaceae**								
*Urtica dioica *L. subsp. *dioica *MEB7	Ortica, 'rdica	Fresh cut aerial part	As food with corn	'German measles' in Turkeys ("When they go bald")	3 (PI)	No		
		Aerial part	Infusion (compresses)	On bruises	1 (PI)	No		
Valerianaceae								
*Valeriana officinalis *L. MEA10	Vallariena	Root or leaves	Decoction (e.u.)	To disinfect wounds in mules	1(PE)	No		
**Vitaceae**								
*Vitis vinifera *L. LU32	Vite	Vermouth or Marsala	With water (u.i.)	Given to sick turkeys to drink	4(PI)	No	Al	

**Abbreviations**: **Localities**: AG = Agnone; CA = Castiglione di Carovilli; CP = Capracotta; PE = Pescolanciano; PI = Pietrabbondante **Way of use**: e.u. = external use **Period of gathering **Su = summer; Al = always **Habitat **Daw = damp woods; He = hedges; Ru = ruins; Unc = uncultivated areas; Wa = walls; Wo = woods.

**Table 2:** Animal, plant and mineral derivatives in folk medicine

**Agent**	**Method of employment and use**	**Bibliographical use (references)**	**Field use (frequency of citation)**
***Badger***	Coat, with splinters of brass under the halters of the horses to protect them from the evil eye	Conti [2]	
*Hare's blood*	Pneumonia and pleurisy. It is kept dried, dissolved in lukewarm water (internal use)	Moffa [4]	
	Spread on painful joints, especially the shoulder		1(PI)
*Cinders*	On potato plants to protect them from parasites		4(PI)
*Cobwebs*	Applied fresh to wounds as an haemostatic and cicatrising agent	Moffa [4]	1(PI)
*Domestic mouse (!!)*	As food for the incontinence, skinned and cooked		6 (PI)
*Ass Dung*	Dried and powdered on bleeding wounds	Moffa [4]	
*Egg white*	Whisked until stiff, it was mixed with lime and wrapped on fractured limbs as plaster		4 (PG)
	For sprains and haematomas, whisked until stiff, applied to the skin and bound		4 (CH) 4 (VG)
*Fat*	Fat of fox or horse applied to pimples to bring them to a head	Conti [2]	
	Hen fat spread on sores of the neck of oxen		4 (PI)
*Hard eggs and March "ricotta"*	As food in cases of dysentery	Conti [2]	
*Leeches*	Applied to the skin against typhus		2 (CH)
*Salt water*	External cysts, the skin was always kept wet		4 (PI)
*Snail ("ciammarùca")*	The mucilage was applied to serious skin inflammations		3 (PI)
*Soot*	De-wormer for children, dissolved in water (internal use		4 (VG)
	On wounds as an anti-parasitic (veterinary use)		4 (VG)
	As a repellent, with wood cinder in molehills		6 (PI)
*Sl oughing of snake (old skin)*	Crumbled and mixed with food: for women with difficult pregnancy (magic use)		4 (CH) 6 (PI)
	Put in a small bag as an amulet against evil eye		4(PI)
*Wax of ear*	On pimples (applied with *Hyoscyamus *sp. leave)	Pierro [3]	

With the aim of highlighting new pharmaco-botanical aspects, data relating to the medicinal uses of plants were compared with Italian and European pharmacobotanical and ethnobotanical reference works (Gastaldo [22]; Boni and Patri [23]); Cappelletti [24]; Bellomaria and Della Mora [25]; Guarrera [26]; Uncini Manganelli and Tomei [27,28]; Pieroni [29]; Ballero et al. [30]; Leporatti and Corradi [31]; Pieroni et al. [32,33]; Atzei [34]; Viegi et al. [35]; Guarrera et al. [36,37]; Scherrer et al. [38] (and references therein); Tammaro and Xepapadakis [39]; Gonzàles-Tejero et al. [40]; Bonet et al. [41,42]; Novaretti and Lemordant [43]; Raja et al. [44]; Vàzquez et al. [45]; Pieroni et al. [46]; Agelet and Vallès [47].

## Results and Discussion

The results of our research are shown in the Additional file 1 and in Tables 1, 2. Information was collected relating to 70 taxa belonging to 39 families. In total, 64 plants are used in human therapy, 5 as insect repellent and anti-parasitical agents, 11 in veterinary medicine, 1 for preserving eggs and cheese and 4 for magical uses.

The family most frequently represented is that of the *Compositae *with 8 taxa. On the basis of the research conducted, it can be seen that the plants used most often (in terms of the number of people who cited them and/or the number of uses to which they are put) are: *Vitis vinifera *L.; *Urtica dioica *L. subsp. *dioica*; *Malva sylvestris *L. subsp. *sylvestris*; *Juglans regia *L.; *Abies alba *Miller; *Allium sativum *L.; *Olea europaea *L; *Cynodon dactylon *(L.) Pers.; *Triticum aestivum *L.; *Ficus carica *L.; *Matricaria chamomilla *L.; *Rubus ulmifolius *Schott.

The most complete and exhaustive interviews were obtained from women (housewives and farmers) who listed particularly home remedies and plant medicines derived from species cultivated in kitchen-gardens and fields.

The parts of the plants most used for medicinal purposes, according to the number of citations are, in decreasing order: fruits and seeds; leaves; aerial parts; rhizomes and tubers; entire plant; bulbs; resin; flowers (including flower heads). External uses predominate over internal by about 60–40. Decoction – almost always in water – is the main method of preparation for oral administration, while poultices and direct application of the plant are the most important methods for topical use.

Nine plant preparations are administered as food-medicine.

Eighty-two uses are reported by at least three informants among medicinal practices, whilst there are 8 anti-parasitic uses.

Most pathologies treated with plants are dermatological (29%), digestive (17%), respiratory (15,5%) and renal (4,1%) illnesses.

Field data show that among the total uses, only 25% are now still practised.

Concerning the habitat, the most frequently used plants are gathered in uncultivated areas, near ruins and damp sites. A consistent number of plant species (24) are/were cultivated in kitchen-gardens and fields (some of these are also naturalized plants in the area).

### Ethnobotanicity indexes

The ethnobotanicity index (E.I.) according Portères (ratio between the useful plants and the total flora, expressed as a percentage) [48] is 6 % for medicinal plants (9,75 % if referred to all the useful plants according unpublished data of the authors). This means that 6 % of the plants are known to be useful in folk medicine. Comparing this index with that of other small territories [42] (data obtained from the authors mainly with Pharmacy Degree Theses realized in Spain) we can observe that the degree of knowledge of medicinal plants (in folk medicine and veterinary science) and useful plants in the upper Molise region is lower than in the Spanish region of L'Alt Empordà (11'%), considered provisional by the authors since the area was not completely studied from the ethnobotanical point of view. The indexes of the upper Molise are similar to that of Córdoba (8.8%), but much lower than those of Les Guilleries (20%), Caurel (27.9%) and a further three Spanish territories [42]. We also compared the E.I. of the upper Molise region with that of other Italian areas (Friuli-Venezia Giulia region [49], Majella (Abruzzo) [50], Latium region [26], Acquapendente (Latium) [36,51], Maratea (Basilicata) [37,52,53]) and of Italy as a whole [54]. These values were obtained by dividing the number of species cited in folk medicine and veterinary science (or in all sectors of ethnobotanical knowledge) of each area by the number of species of the respective floras [21,55,56]. We can note (see Table 3) that the values of E.I. in the selected Italian areas are between 3.83 % and 10,75 % (upper Molise, Acquapendente, Maratea) [36,37,51-53]. Instead, the values for the entire country [54] are higher (from 14.24 % to 17.57 %). These Italian values of E.I. are generally lower than the corresponding ones for Spanish, perhaps due to a more rapid process of cultural erosion in Italy. Nevertheless, it appear that the indexes of the Spanish areas were calculated, according Portères, on all the plants used by the informants or at least on the plants known and for which the informants gave a name, and not only on medicinal plants (J. Vallès, personal communication). Complete folk knowledge of plants is greatly appreciated in scientific ethnobotanical research in Spain, also by pharmacobotanists, while in Italy many aspects of this research are often entrusted to the good will of single researchers.

**Table 3:** Ethnobotanicity indexes (E.I.) concerning vascular plants according Portères [48] for some Italian areas, compared with the E.I. of Italy [54]. References inside parentheses [x].

**A**	**B**	**C**	**D**	**E**	**F**	**G**	**H**
Area	Medicinal plants of folk use (*)	Cultivated plants (**) among the medicinal species of folk use	Plants used in all sectors of the ethnobotanical knowledge	Cultivated plants (**) among the species of all folk uses	Total flora	E.I. $\frac{\text{B}-\text{C}}{\text{F}}\times 100$	E.I. $\frac{\text{D}-\text{E}}{\text{F}}\times 100$
Friuli-Venezia Giulia region	177 [49]	2 [49]	181 [49]	2 [49]	3335 [21]	5,25 %	5,37 %
Latium region	283 [26]	13 [26]	350 [26]	20 [26]	3228 [21]	8,36 %	10,22 %
Acquapendente (Latium)	80 [36]	22 [36]	142 [36,51]	27 [36,51]	1070 [56]	5,42 %	10,75 %
Majella (Abruzzo)	113 [50]	24 [50]	148 [50]	24 [50]	1700 [55]	5,23 %	7,29 %
High Molise region	70 (***)	22 (***)	109 (***)	31 (***)	800 (***)	6 %	9,75 %
Maratea (Basilicata)	53 [37]	14 [37]	125 [37,52,53]	17 [37,52,53]	1019 (****)	3,83 %	10,60 %
Italy	1163 [54]	76 [54]	1458 [54]	117 [54]	7634 [21]	14,24 %	17,57 %

(*) Including plants used in veterinary science (**) Plants at the same time cultivated and wild, or cultivated and naturalized were not calculated (***) This study (****) F. Lucchese, personal communication

### Medicinal uses

The predominance of external over internal uses, and of dermatological pathologies treated in folk medicine of upper Molise can be connected with the many rural activities. In fact wounds, insect bites, blisters etc. (main disturbances in skin diseases) are the most common incidents in field activities and in woodcutters' work. Within the category of skin ailments, wounds represent 48 %, whilst insect bites account for 19%. Digestive disturbances (the second category of illnesses in order of importance) are again quite important considered in the folk medicine of upper Molise, given the large quantity of plants available containing bitter substances, mucilages and essential oils useful in cases of gastritis, colitis, intestinal worms etc. Furthermore, they can be linked to the cold during bad seasons. Respiratory pathologies (the third category of illnesses represented) are connected with the very cold climate in winter.

Amongst the medicinal uses, certain practises would appear to be recorded for the first time (the active ingredients are reported above all by Anzalone [57], Gastaldo [22] and Guarrera [26]). Amongst the most important results of our research we wish to cite: *Lobaria pulmonaria *(L.) Hoffm., thallus applied on cuts, an unreported usage. The plant contains antiseptic lichen acids [57], phenolic substances [58], hydrocarbons, sterols – among which ergosterol, episterol, fecosterol, lichesterol -, fatty acids [59,60] and depsidones [61]; the efficacy is confirmed by its anti-ulcer and anti-inflammatory properties [58,62]. Lichen is one of the plants that is most sensitive to the aerial pollution [63] and it indicates an uncontaminated environment. Therefore areas where this lichen grows could be the more suitable for the cultivation of medicinal plants, small wood fruits etc. *L. pulmonaria *was widely used as a cicatrising agent and antiseptic in the council areas of Pescolanciano, Pietrabbondante and Chiauci. The area is dominated by silver firs (on which this lichen grows) mixed with Turkey oaks and beech trees at higher altitudes. Carbon production (especially from beech) probably once represented an important resource for the villages concerned. Instead, near the council areas of Vastogirardi and Castiglione, on the other side of the mountain, the main activities were agriculture and sheep-farming. In these villages we found no trace of the use of pulmonary lichen, but the limited number of species of moss were used as cicatrising and anti-haemorrhagic agents, as well as a mushroom of the *Lycoperdon *genus (spores). This last rare use is reported by Lomagno and Lomagno Caramiello [64], Atzei [34], Agelet and Vallès [65].

#### *Pulmonaria apennina *Cristof. & Puppi and *Centaurium erythraea *Rafn

The use for bruises can be explained by their containing, respectively, mucilages and gum or wax (the use of *C*. *erythraea *is reported also by Appi and Pagnucco [66]).

#### *Adiantum capillus-veneris *L

The use for haemorrhages [32] could be due to gallic acid and tannins [22], while the mild analgesic action in labour pains would result from anti-inflammatory triterpens such as β-sitosterol, stigmasterol and campesterol [67,68].

#### *Asplenium trichomanes *L

The plant has been reported as demulcent, expectorant and laxative [69], but also to promote menstruation [70]. Caution is nevertheless advisable, because many ferns contain carcinogens [71]. Since the species, according to Gastaldo [22], contains active compounds similar to those of *A. capillus-veneris*, the use for regulating menstruation [72,73] could be due to similar sterols.

#### Rare uses

The emollient properties of the *Abies alba *L. resin for removing splinters find some confirmations [74,75]; an anti-inflammatory use of the resin is sometimes described, e.g. in case of abscesses [42,43,47]. The usage of *Achillea millefolium *L. for toothache was only described for the Alps [76]; it can be explained by the anti-inflammatory proazulene contained in its essential oil. Very rare is the use of *Sonchus oleraceus *L. latex (sesquiterpene lactones, taraxasterol) for insect stings [34], but also unpublished is the same use described for *Chelidonium majus *L., a plant generally employed for warts, although analgesic action is attributed to the plant [77]. Moreover, the inclusion of *Allium sativum *L. in a variety of anti-flu decoctions is particularly interesting (in the folk knowledge no distinction is made between fever due to colds and to viruses); the antiseptic effect is known, while some antiviral properties have been recognized [78]. Other rare uses are: *Saccharomyices cerevisiae *Rees., thallus on abscesses (proteins, vitamins of the B group), generally antiseptic in the intestine; *Cyclamen hederifolium*, tuber for chilblains (saponin).

In the field of phyto-therapy, certain species may be considered as particularly representative. Concerning *Malva sylvestris *L. subsp. *sylvestris*, a proverb suggests that: *a "mmàleve d'ogni mmale salve" *(mallows prevents all ills), and that the name itself means "evil (or ailment) go" (it is used in a large number of preparations).

In the isolated conditions caused by long winters and heavy snowfalls, major and most frequent use was made of: oil, bread, wine and vinegar (used in the home-made treatment of many ailments). These derivative products are generally not taken into consideration by the main texts of pharmaco-botany, whilst they are reported by various ethnobotanical local works.

#### *Triticum aestivum *L

The parts of the grain used were: bran (toothache, rheumatism) and warm kernels (bronchitis, spots and boils). Small tablets were made with bread that were conserved to be used with various plants. The bread cooked in milk was used on bruises or, mixed with saliva and sugar, applied to cuts. In one case it was successfully applied to a finger to treat the initial phases of gangrene.

#### *Vitis vinifera *L

and derivatives Wine is used on cuts, to treat bronchitis by inhalation, for footbaths (this last is an uncommon use), and to cure rheumatism. Vinegar poultices were used to treat fever and high temperatures. The cooked must and green grape pulp helped in cases of severe diarrhoea.

#### *Olea europaea *L

'Olio ferrato', was prepared by heating olive oil in a frying pan, usually together with three nails: this ointment was used both in human and in veterinary medicine to cure wounds. Hot oil served for earache and to massage forearms in case of swollen glands for sore throat and fevers. Beeswax was warmed near a lamp to form an ointment applied in case of burns.

The widespread use of these derivative products typical of Mediterranean areas also testifies to the degree of simplicity and primitiveness of the folk medical culture and the essential nature of the local knowledge.

### Veterinary uses

Amongst the most important veterinary uses (see Table 1) we quote: *Valeriana officinalis *L., a decoction of roots and leaves (new use) or oil mixed with soot for treating wounds in mules; *Urtica dioica *L subsp. *dioica*, the leaves used as fodder for turkeys suffering from rubella and an infusion applied with poultices for bruising in cattle; olive oil, clay and sugar for repairing fractures horns in cattle; stalks of burnt wheat mixed with olive oil and applied to cuts and wounds [79]. Almost all the uses in this field have nowadays been abandoned.

### Food preservation and anti-parasitic uses

We wish to emphasise the widespread knowledge of the following uses, which were mentioned in almost every single interview (see Additional file 1).

#### *Capsicum frutescens *L

crumbled, dried berries were used to protect against parasites in kitchen containers, especially those containing herbs. Major and minor capsaicinoids [80] have been characterized in this plant. Atzei [34] reports the use of dried chilli peppers as a mosquito repellent; repellent properties of *Capsicum *extracts were described also by Nolte and Barnett [81] and Antonious et al. [82].

#### *Allium sativum *L

cloves (essential oil) placed in cases of legumes and grain as repellent.

An early use to prevent woodworm in beehives had already been recorded [50] and its use as a fly and mosquito repellent [34,83]. Other confirmations are found in Valerio and Maroli [84] and in Bhuyan et al. [85].

#### *Juglans regia *L

the anti-parasitic use (naphtoquinone compounds) was described for other Italian regions [86].

#### *Triticum aestivum *L

kernels used in grain crates for keeping cheese and eggs fresh, for up

to six months from the onset of summer [2]. The effect could be due to a maintenance of standard temperature caused by the accumulation of the grains.

There is also an interesting comparison between the manner in which different plants are used in the same way in different regions. The plants in question are the aquiline fern (*Pteridium aquilinum *(L.) Kuhn.) in Molise, and the black elder (*Sambucus nigra *L.) [87] in Abruzzo, the parts used being, respectively, the fronds and inflorescence. Both are immersed in whey and hung up in houses to attract and trap flies, the plants then being thrown out (here the plants seem to function merely as a base, the whey representing the actual "vegetable" insect-trap). A similar use in described in Abruzzo with *Glycyrrhiza glabra *L. [83], a plant that has sticky glands.

### Beliefs and rituals

Amongst the most interesting ritual uses reports we find *Clematis vitalba *L., branches of which were wound 7 times around the necks of sheep which appeared to be particularly nervous (a similar use was described by Atzei [34]) and broom (*Spartium junceum *L.) and silver fir (*Abies alba *Miller) for making the characteristic torches carried during the traditional " 'ndocciata". The magical use of a sorghum broom is also described by Conti [2]. "Ferrato", or "iron" oil used to be made from olive oil usually cooked in a frying-pan with three nails: the unguent was used on both humans and animals for treating cuts, burns and earache (in these recipes the ritual use of iron was combined with the curative properties of the oil). The oil was rubbed into the forearm to cure throat inflammations, combined with beeswax it was used for treating scalds and, as a paste, for itchiness. Other medicinal uses are also connected with rituals. For example, a decoction of 3 roots of mallow, 3 of nettle and 3 of coltsfoot was used for stomach-ache, the ritual use of 3 being introduced into the recipe, just as the use of an odd number of each ingredient (1–3–5–7, etc.) is important in others cases.

At the end of the winter, in the period of Easter, young girls of Molise used to ask the "blessed palm" (*Olea europaea *L.) for their beloved, the leaves then being thrown on to burning coals for a sign or divination [2].

The presence of many rituals linked with officinal uses, and of several agricultural feasts of the grain [18], is a remainder of the pagan culture of the Romans and of even earlier populations.

A small archaeological bronze tablet ("Tavola Osca"), now kept in the British Museum in London, illustrates this agricultural background of the local culture. The tablet was found near the Monte del Cerro, between Capracotta and Agnone, in the place named "Uorte", that is to say kitchen garden (Hortus in Latin language and Hùrz on the tablet). We can learn from this very important tablet, dating back to 250 B.C., that the place was devoted to Kerrer (the goddess Cerere). According to this tablet, a sacrifice was made to four divinities near a sanctuary at the time of the Floralia feasts. On the tablet, several agricultural divinities are named, including: Cerere; Persefone (daughter of Cerere); Maia, Italic goddess of the spring; Flora, protector of the shoots) [88].

Besides, over two hundred years ago, the Molise region came under the spiritual administration of Benevento, a centre of ritual and witchcraft (see the famous Benevento walnut, around which witches use to dance). This influence was undoubtedly extremely strong in villages of the Molise, to the extent that Cardinal Orsini, later Pope Benedict XII, condemned certain superstitious practices in his LI edict, these practices being listed in Moffa [4].

### Animal, plant and mineral derivatives in folk medicine

During the research activity, certain questions were asked during the interviews relating more generally to the animal world so as to obtain information that can help us have at least a partial understanding of the ethno-biological customs of this region. Further data was obtained from the papers consulted [2-4,89]. Certain vegetable (ash and soot) and mineral uses are also reported (see Table 2). Some plant-derived products were not included in the main table (Additional file 1) for these reasons: a) cinders come from undetermined plants b) the information on the earwax applied with a *Hyoscyamus *sp. leave comes from a bibliographical reference and not from field research.

## Conclusion

This survey was carried out in an ample research project for the study of the ethnobotanical resources of Italy [26,35-37,50-54,86,90-93].

The predominance of external over internal uses, and of dermatological pathologies treated in the folk medicine of upper Molise can be connected with the still now rural culture of this area, where there is little industrial activity.

The research proves that the uses in veterinary science have been abandoned, and that the remedies of folk medicine are not very widely practiced today (only 25% of the total uses still continue). Nevertheless, the fact that eighty-two uses are reported by at least three informants among medicinal practices, whilst there are also 8 anti-parasitic uses, shows that there is still today a large consensus regarding this folk knowledge.

A certain quantity of new or rare uses were found, related to *Lobaria pulmonaria*, *Pulmonaria apennina, Centaurium erythraea, Sonchus oleraceus *etc. These novelties testify to the wealth of the local plant medicine. Some medicinal plants and small wood fruits could be cultivated in the large areas without pollution of the investigated area (see *Lobaria pulmonaria*).

Medicinal uses are sometimes still now bound to rituals, esteemed important in the past to reinforce the psychological efficacy of a plant drug. These rituals are connected with the primordial culture of the ancient populations. Animal and mineral derivatives enrich the inventory of local folk medicine remedies.

The values of E.I. (ethnobotanicity index) of the high Molise region for medicinal plants are among the highest in Italy, perhaps due to the geographic and cultural isolation of the studied area. Nevertheless they are much lower than those in Spanish areas, perhaps due to a more rapid process of cultural erosion that in Italy advanced more quickly that in Spain.

The consideration that the more frequently used plants grow in uncultivated areas, near ruins and damp sites, or in kitchen-gardens and fields, but not in woods (except for *Abies alba*), where men typically worked, leads us to consider once again the important role played by women in this field, as had already emerged from the interviews.

In the high Molise it was found that, within the four walls of the home, the principal role was played by the women, who were entrusted with almost all the tasks, whilst the men generally conducted their activities outside. It was also the woman's responsibility to treat minor ailments, mainly by means of decoctions which may vary from house to house although the basic composition was always the same.

The research conducted revealed a deep-rooted and widespread habit of husbanding the family's resources, mainly as a result of the isolation in which so many live. As we have already noted, the Upper Molise has a particularly cold and damp climate which still today can cause severe difficulties, especially during the winter months when there are heavy snowfalls even at low altitudes. This physical isolation has resulted in two major and onerous factors both for the individual family nucleus and for the community itself: 1) *supplies *are practically throughout the period of snowfalls. 2) *preservation *of foodstuffs, in particular grain and legumes. These, preserved in large quantities, were often damaged by worms and insects which could severe hunger for the entire family.

Whilst these factors contributed to the widespread knowledge of means of conserving foodstuffs, they also led to the use of products easily available within each home.

## Competing interests

The author(s) declare that they have no competing interests.

## Authors' contributions

The field work for data collection was carried out above all by SM. Data analysis was conducted by all authors. The manuscript was prepared by PMG. All authors read and approved the final manuscript.

## Supplementary Material

Additional file 1 Medicinal, anti-parasitic and ritual uses of plants in the high Molise region. The data provided concern mainly medicinal and anti-parasitic uses of plants collected during field interviews in the high Molise region. Some ritual uses of plants are also described. Click here for file
