# Supplementary material for: Ethnophytotherapeutical research in the high Molise region (Central-Southern Italy)
Source: J Ethnobiol Ethnomed. 2008 Mar 11;4:7. doi: 10.1186/1746-4269-4-7 (PMC2322956; doi:10.1186/1746-4269-4-7)
Supplement: Additional file 1 — Medicinal, anti-parasitic and ritual uses of plants in the high Molise region. The data provided concern mainly medicinal and anti-parasitic uses of plants collected during field interviews in the high Molise region. Some ritual uses of plants are also described. [file 1746-4269-4-7-S1.pdf]

Additional file 1. Medicinal, anti-parasitic and ritual uses of plants in the high Molise region

Family, scientific name, voucher specimen	*Vernacular names*	*Parts used*	*Preparation*	*Local uses*	*Number of informants and locality*	*Present use*	*Period of gathering*	*Habitat*
**FUNGI**								
**Lycoperdaceae**								
Lycoperdon sp.	Véscia d' lùpo	Spores	Fresh spores on open wounds	As anti-haemorrhagic and antiseptic	8 (VG)	No	Sp-Su-Fa	Mopa
**Saccharomicetaceae**								
Saccharomices cerevisiae Rees.	Lievito	Thallus	Mixed with water to form a cream	Applied on gum abscesses to "ripen" them	4 (VG)	No	Al	-
**MUSCI**								
Musci sp. pl.		Entire dry thallus	Crumbled without hearth	As anti-haemorrhagic and cicatrising agent	4 (VG)	No	Sp-Su-Fa	Wo
**LICHENES**								
**Stictaceae**								
Lobaria pulmonaria (L.) Hoffm. - MEB10	Lichene polmonario	Fresh or dry thallus	Chewed or crumbled on wounds (e.u.)	As a cicatrising agent and antiseptic	8 (PI): fresh plant; 3 (PE): chewed	Yes (rare)	Al	*Abies alba* trunks
**PTERYDOPHYTA**								
**Equisetaceae**								
Equisetum sp.	Cùdìlle(AG) Codella (PE)	Aerial part	Decoction, up to 1lt./day (i.u.)	To treat kidneys after surgical operation (as a depurative)	1 (AG)	No	Al	Da, Shpl
	Cudìlle(PI)	Aerial part	Decoction	As a diuretic	2 (PI)	No	Al	
**Adiantaceae**								
Adiantum capillus-veneris L. - LU1	Pil'evenere	Entire plant	Decoction (i.u.)	To stop haemorrhages after childbirth	6 (PI)	No	Al	Da, we
	Pile e' Venere	Entire plant	Decoction (i.u.)	To soothe the pain in childbirth	1 (PE) 1 (PI)	No		
**Aspleniaceae**								
Asplenium trichomanes L. – MED5	Felce	Aerial part	Infusion	To regularize menstruation	2 (CH)	No	Al	Rock
Ceterach officinarum Willd. – MED4		Aerial part	Infusion with chamomile	After childbirth	1 (PI)	No	Al	Rock, wa

**Hypolepidaceae**								
Pteridium aquilinum (L.) Kuhn. subsp. *aquilinum* – MED6	Felce	Aerial part	Dipped in milk whey and hung in house	To attract flies that stick to it; then the fern is thrown away	6 (PI)	No	Al	Da, shpl
	Felce	Fresh aerial part	Put cross-shaped on the abdomen	For intestinal pains	2 (PI)	No		
		Aerial part	Hung in the house	To remove flies	5 (PG)<br>2 (PS)	No		
**GYMNOSPERMAE**								
**Pinaceae**								
Abies alba Miller MEC6	Abete	Resin	Heated (e.u.)	To cicatrise bleeding wounds	4 (PI)<br>4 (VG)<br>1 (PE)	No	Sp-Su	Wo with *Quercu s cerris*
		Resin	Applied mildly hot and left to cool (e.u.)	Insect bites when the sting remains in the skin	8 (PI)<br>4 (VG)<br>2 (PE)	No	Sp-Su	and *Fagus*
		Resin	External use	Rheumatic pains	1 (PI)<br>2 (CH)	No	Sp-Su	
		Resin	Put in bowling water at first (i.u.)	Constipation and related pains	1 (AG)	No	Sp-begin-ning Su	
		Resin	Burnt in houses or rooms of patients	To purify the air	4 (VG)<br>2 (PI)	No	Sp	
		Resin	Heated in a bain-marie, bandages then soaked in it	Applied to fractured limbs as a plaster	4 (VG)<br>2 (PI)	No	Sp	
**ANGIOSPERMAE**								
**Boraginaceae**								
Pulmonaria apennina Cristof. & Puppi – MEB9		Leaves	Decoction: poultice on painful parts	Illnesses of blood circulation	4 (VG)	No	Al	Unc, da
**Caprifoliaceae**								
Sambucus nigra L. MEA14	"Panerìcia" (inflorescence)	Flowers	Infusion (i.u.)	Coughs, bronchitis	2 (VG)<br>1 (PE)<br>3 (PI)	No	Sp-Su	Ro, coh
Sambucus ebulus L. MEA15	Jèbbs (VG) Palmùnere (PE)	Leaves	Entire fresh leaves placed in shoes	To prevent blisters and as a refreshing agent (e.u.)	3 (CA)<br>1 (PE)<br>4 (VG)	No	Sp-Su-Fa	Ro, ru.
**Compositae**								
Achillea millefolium L. MEB3	Millefoglio	Entire plant	Infusion (also decoction) (e.u.)	Gargles / mouthwashes for toothache	2 (AG)	No	Sp-Su	Da, me

		Flower heads	Infusion (i.u.)	To provoke menstruation	1 (PE)	No	Sp-Su	
Artemisia absinthium L. – MEA12	Assenzio	Aerial part	Rubbed onto the skin	To remove horse-flies and mosquitoes	1 (CP)	Yes	Al	Ru, he
Helminthotheca echioides (L.) Holub LU3	Spràina bianca	Leaves	Poultice chewed and crushed (e.u.)	On wounds	2 (PI)<br>2 (PG)<br>1 (CP)	No	Sp-Su	Unc, dra
Lactuca sativa L. LU2	Insalata	Leaves	Cooked with "Strega" liqueur "	On painful or inflamed gums	2 (PI)	No	Al	Cu
Matricaria chamomilla L. – MEA11	Campomilla	Flower heads	Infusion with *Ceterach officinarum*	After childbirth	1 (PI)	No	Al	Ru, kigd
		Flower heads	Infusion applied as enema	To clean the intestine	1 (PE)	No		
		Flower heads	Infusion (i.u.) with sugared almonds always odd numbers (1,3,5…)	For colds	2 (AG)	Yes		
		Flower heads	Infusion	Intestinal pains	4 (PG)	Yes		
		Flower heads	Infusion (e.u.)	Vaginal washes and also in case of inflammation	4 (PG)	No		
Petasites hybridus (L.) Gaertner, B. Meyer et Scherb. subsp. *hybridus* – MEE2	Cuoppazz'	Leaves	Fresh entire leaves in summer	Worn in pants and shoes and on sores as refreshing agent	4 (VG)	No	Su	Da, di
Sonchus oleraceus L. LU4	Cacigne	Leaves	In poultice boiled and mixed with bread crumbs (e.u.)	For insect and snake venom	1 (PE)	No		
Tussilago farfara L. MEA13	Cuoppo	Leaves	Infusion (i.u.)	Coughs	1 (PI)	No	Sp-Su	Ro (on
		Rhizome	Decoction (3 roots of *T. farfara*, 3 of *Malva*, 3 of nettle)	Stomach pains	2 (AG)	Yes	Al	clayey soils)
**Cruciferae**								
Brassica oleracea L. LU5	Verza	Leaves	Poultice cooked over embers and mixed with the hands	Applied on wounds and bruises	8 (PI)	No	Al	Cu
**Dipsacaceae**								
Dipsacus fullonum L. MEA1	Cardo	Aerial part	One plant was placed inside and another outside the window (divination)	According to which plant flowered, a girl of marriage-able age would marry a fellow townsman or a stranger	4 (PI)	No	Before the flowe-ring	Dra, unc
		Aerial part	As above but the inflorescence was singed	Divination	2 (AG)	No		
**Euphorbiaceae**								

Euphorbia lathyris L. MEE1	Tòtemaje Tetemaglie	Entire plant	Planted in kitchen-gardens	It removes moles	1 (PE) 2 (PI)	Yes	-	Me, kigd (cu), unc
**Gentianaceae**								
Centaurium erythraea Rafn. – MEA9		Rhizome, entire plant	Decoction (e.u; .i.u.)	Compresses on bruises, pains, dislocations; fever, anti-inflammatory (i.u.)	6 (PI)	Yes Rare	Sp-Su	Cl, ro, mopa
		Rhizome	Decoction (i.u.)	Fever	1 (PI) 2 (PS)	Yes		
Gentiana verna L.	Genzianella	Plant in bloom	Infusion (i.u.)	Depurative	4 (VG)	No	Sp-Su	Mopa
**Graminaceae**								
Cynodon dactylon (L.) Pers. – LU6	Gramegna, gramigna	Entire plant including rhizome	Decoction (i.u.)	Intestinal burnings	2 (PI) 4 (VG)	Yes	Su-Fa	Unc, kigd, vi
		Rhizome	Decoction (i.u.)	Diuretic, for kidney stones	3 (CH) 2 (PI)	Yes	Su-Fa	
		Rhizome	Decoction (i.u.)	Depurative	2 (AG)	No	Su-Fa	
		Rhizome	Decoction (i.u.)	Diuretic, depurative	2 (PE) 9 (PI)	Yes	Su-Fa	
		Rhizome	Decoction (i.u.)	For nervous complaints ("Panacea")	1 (PE)	Yes	Su-Fa	
		Rhizome	Decoction (e.u.)	Washes to prevent hair loss	6 (PI)	No	Sp-Su-Fa	
		Rhizome	Decoction (i.u.)	Cystitis of women	2(PI)	No	Sp-Su-Fa	
Hordeum vulgare L. – LU7	Orzo	Caryopsis	Roasted fruits were applied hot on the chest enveloped in a cloth	In case of respiratory diseases (e.g. bronchitis)	1(PI)	No	Al	Cu
Sorghum bicolor (L.) Moench – LU8	Saggina	Stems	The broom was put behind the door of the house often with corncobs (magical use)	It was believed that witches, before entering, had to count the threads of the broom and grains of maize which would take all night	4 (PS) 2 (PI)	Yes	Al	Cu
Triticum aestivum L. – LU9	Grano	Caryopsis	Fruits were roasted and applied hot to the chest enveloped in a cloth or in a small cloth bag	In case of respiratory ailments (e.g. bronchitis)	3 (PI) 4 (VG) 4 (PG)	No	Al	Cu
		Caryopsis	Poultice with hot water (e.u.)	Sore throat	4(VG)	No	Al	

		Pericarp of the fruit	Roasted bran applied to the skin	Rheumatic pains	2 (PI)<br>4 (VG)	No	Al	
		Bran	Compress	Pimples and pustules	4 (PI)	No	Al	
		Caryopsis	Hens eggs and cheeses placed in large cases of wheat at the start of summer	To preserve eggs and cheeses (these would keep throughout the summer)	12 (PI, CA, PG, VG)	No	Al	
		Bread	Poultice with sugar and saliva	Wounds and gangrene (a lady claimed to have healed a serious infection by binding the wound with the preparation)	3 (CH)	No	Al	
		Hot pericarp of the fruit	Compress	Toothache	2 (PI)	No	Al	
Lolium sp.	Gliuòglie	Caryopsis	Seeds were often mixed with wheat	Sedative. If a person is slow, one says " ha mangiato pan de gliuòglie" (he's eaten the plant")	2 (PI)	No	Su	Unc, fi
**Juglandaceae**								
Juglans regia L. MEB8	Noce	Leaves	Macerated with saliva (e.u.)	On pimples and pustules	4 (PI)	Yes rare	Al	Cu
		Leaves	Fresh leaves in cases where flour was kept	Anti-parasitic use, to prevent the formation of worms	4 (PE)	No		
		Leaves	Fresh leaves in cases of corns and pulses	Anti-parasitic agent (insects, worms)	4 (AG)<br>2 (PI)	No		
		Leaves	Fresh leaves	On wounds as an antiseptic	4 (VG)	No		
**Labiatae**								
Calamintha nepeta (L.) Savi – LU10	Menduccia	Leaves	Juice from a handful crushed and strained, with water (i.u.)	Stomach pains, intestinal spasms	2 (AG)	Yes	Al	Unc
Mentha longifolia(L.) Huds. MEA5	Menta	Leaves	Infusion (i.u.)	Stomach pains	1 (PE)	No	Al	Da
Salvia officinalis L. LU11	Salvia	Leaves	Fresh rubbed leaves	To clean and disinfect teeth / gums	2 (PI)<br>1 (PS)	No	Al	Ru, kigd (cu)
**Lauraceae**								

Laurus nobilis L. LU12	Lauro	Leaves	Infusion (i.u.) with apple, chamomile, garlic (always odd numbers!)	Fever, tussis, cold	2 (AG)	No	Al	Tewo
		Dry leaves	Decoction with fennel seeds and half a lemon	Digestive	6 (PI)	No		
**Leguminosae**								
Cicer arietinum L. LU13	Ceci	Seeds	As food<br>(to be eaten in plenty)	In case of kidney stones	1 (PI)	Yes	Al	Cu
Phaseolus vulgaris L. LU14.	Fasciuòle	Seeds	"Scattone". Hot dish with homemade pasta and wine	Diet for coughs or bronchitis	6 (VG)	No	Al	Cu
**Liliaceae**								
Allium sativum L.	Héaglie	Bulb	Fresh crushed cloves with salt (e.u.)	For chilblains	3 (PI)	Yes rare	Al	Cu
LU15		Bulb	Entire cloves	Repellent agent on sacks of pulses	6 (PI)	No		
		Bulb	Fresh crushed cloves	For chilblains	4 (CH)	Yes		
		Bulb	Cloves sewn to make a necklace (e.u.)	To eliminate worms	4 (CH)<br>3 (AG)<br>8 (VG)	No		
		Bulb	Crushed and put in a compress on the abdomen	To eliminate worms	1(PE)	No		
		Bulb	Roasted, then rubbed on the abdomen	To eliminate worms	1 (PE)	No		
		Bulb	As food	To regularize blood pressure	4 (PI)	Yes		
Allium cepa L. LU16		Bulb	As food	Refreshing for the intestine	1 (PE)	Yes	Al	Cu
Allium ascalonicum L. – LU17	Scalogno	Bulb	As food	"To purify the blood"	1 (PI)	No	Al	Kigd (cu)
**Linaceae**								
Linum usitatissimum L.	Lino	Seeds	Crushed, mixed with olive oil and warmed until small lozenge is formed (e.u.)	Hot poultice with gauze on wounds or in case of bronchitis	4 (PG)	No	Al	Once cu
		Seeds	Warmed over embers	Put on the chest in cases of bronchial catarrh	2 (PI)	No		
**Malvaceae**								
Malva sylvestris L. subsp. *sylvestris* MEA4	Maula	Aerial part	Decoction	Constipation	2 (PI)<br>2 (PE)<br>4 (PG)	Yes	Sp-Su	Unc

		Aerial part	Infusion	Depurative, anti-septic	4 (CH)	Yes	Sp-Su	
		Aerial part	Infusion	Intestinal pains	5 (VG)	No	Sp-Su	
		Aerial part	Infusion for washes (often with chamomile)	Intimate female hygiene also after childbirth	4 (CH)<br>3 (PI)	No	Sp-Su	
		Aerial part	Infusion for gargles	Mouth inflammations	4 (CH)<br>4 (PI)	Yes	Sp-Su	
		Rhizome	Recipe, see *Tussilago farfara*	Stomach pains	2 (AG)	Yes	Al	
		Leaves	Poultice with decoction and bread crumb	Wounds	1 (PE)<br>2 (PI)	No	Al	
		Aerial part	After boiling as food (i.u.)	In case of constipation	4 (PG)	No	Al	
		Dry flowers	Decoction with dried figs (e.u.)	Wounds	1 (PI)	No	Sp-Su	
		Leaves	Boiled and seasoned with lemon as food	To promote expulsion of the placenta	1 (PI)	No	Sp-Su	
**Moraceae**								
Cannabis sativa L.		Fibers ('stoppa')	Poultice with albumen of egg as plaster; hardens as it dries	Often worn for various months	1 (PE)<br>6 (PI)	No	Al	Once Cu
Ficus carica L.<br>LU18	Ficura	Latex	External use	On insect bites (bee stings)	4 (PI)<br>1 (PE)<br>2 (CH)<br>1 (AG)	Yes	Al	Cu
		Dry syconiums	Decoction with pieces of quinces, chamomile etc.	For fever (flu)	7 (PI)<br>1 (CA)	Yes	Al	
		Epicarp of the syconium	Macerated for one night in water; then drunk on an empty stomach in the morning	To facilitate digestion and treat constipation	4 (CH)	No	Su	
		Dry syconiums	Put in must to boil with apple and honey for a few minutes (i.u.)	Bronchitis	4 (PI)	No	Al	
**Oleaceae**								
Olea europaea L.	Olivo	Oil	To massage forearms	Swollen glands (sore throat, fevers)	5 (PI)	Yes	Al	Cu
LU19		Oil	Hot oil (e.u.)	For earache	3 (CH)<br>2 (PI)	Yes		
		Oil	Warmed in frying pan ('olio ferrato')	Applied to small wounds	6 (PI)	No		
		Oil	Poultice	Itching, also of intimate parts	4 (PI)	No		

		Oil	Beeswax warmed near a lamp to form an ointment	Burns	4 (PG)	No		
**Papaveraceae**								
Papaver rhoeas L. subsp. *rhoeas* LU20	Papambre	Leaves and petals	Infusion	Sedative for children	4 (CH)	No	Sp-Su	Unc, fi
		Petals	Infusion	Sedative	2 (AG)	No		
Chelidonium majus L. MEA3	Erba porra	Latex	Fresh latex (e.u.)	Sores, warts, insect bites	3 (CA)<br>3 (CH)<br>1 (AG)<br>4 (VG)	Yes	Al	Ru, shpl
**Plantaginaceae**								
Plantago lanceolata L. MEB2<br>*Plantago major* L. MEE3	Lingua di cane	Fresh leaves	External use	Cicatrizing agent for wounds	2 (PE)	No	Al	Cl, ro, me
**Primulaceae**								
Cyclamen hederifolium Aiton		Tuber	Rubbed fresh tuber	Chilblains	4 (PI)	No	Fa	Shpl
**Rosaceae**								
Crataegus monogyna Jacq. – MEC10	Ceracèlla	Flowers	Infusion (i.u.)	For tussis and in various recipes	4 (PI)	No	Sp	He
Malus domestica (Borkh.) Borkh. – LU21		Fruits	Boiled in must with dried figs and honey for a few minutes (i.u.)	Bronchitis and serious respiratory ailments	4 (PI)	No	Al	Cu
Prunus dulcis (Miller) D.A. Webb – LU22		Exocarp	Decoction (i.u.) with walnut shells and dried figs	Tussis and bronchitis	4 (PG)	No	Al	Cu
Rosa canina L. MEC8	Cacavàsce	Fruits	Infusion	As a sedative tea	4 (VG)	Yes (rare	Su	Pa, he
Rubus ulmifolius Schott MEC11	Ruvètaele Moricole	Leaves	Heated in a grill near embers (e..u.)	Applied to small wounds	4 (PI)	No	Al	He, Unc
	(the fruits)	Leaves	Fresh or chewed with bread and salt	Applied to small wound as a haemostatic	3 (AG)	No		
		Leaves	Fresh leaves	Applied to small wounds	2 (PE)	Yes		
		Leaves	Fresh leaves	Applied on abscesses	4 (PI)	No		
		Leaves	Decoction with dried figs and quinces (i.u.)	Tussis	1 (PI)	No		
**Rutaceae**								

Citrus limon (L.) Burm. LU23		Epicarp of the fruit	Boiled for a long time with orange peel, bramble leaves, chamomile and dried figs to form a syrup	To treat tussis and bronchitis	2 (PI)		Al	Cu
Ruta graveolens L. LU24	Ruta	Leaves	Placed on the ground in the house	To remove mice	4 (CH)	No	Al	Dra, rock
**Scrophulariaceae**								
Verbascum thapsus L.	Zampa dell'orso	Leaves	Fried in olive oil (poultice applying the cooked oil)	On swellings and bruises	3 (CH)	No	Sp-Fa	Dra
		Leaves	Decoction (e.u.)	Inflamed tendons	4 (VG)	No	Al	
**Solanaceae**								
Capsicum frutescens L. LU25	Peperoncino	Fruits	Entire dry or crumbled fruits	In jars of herbs or pulses as anti-parasitic agent	6 (PI)	No	Su	Cu
Solanum tuberosum L. LU26	Patàne	Tuber	Slices of the fresh tuber (e.u.)	Burns	4 (CH)<br>4 (PI)<br>1 (PE)	Yes	Al	Cu
**Umbelliferae**								
Foeniculum vulgare Miller LU28	Finocchiello	Seeds	Decoction	As a digestive and for intestinal pains	2 (PI)<br>1 (PS)	No	Su	Ru, me
Petroselinum crispum (Miller) Fuss – LU29	Petrusìne	Stem without leaves	Placed in the anus as a mechanical clyster for babies	Laxative (CA,CH), with a little soap or olive oil (PI, AG)	3 (CA)<br>4 (CH)<br>6(PI)<br>2(AG)	Yes	Al	Cu
		Leaves	Ptisan (one informer said pregnant women must not eat it)	To provoke abortion	6 (PI)	No	Al	
**Urticaceae**								
Parietaria officinalis L. LU30<br>*Parietaria judaica* L. – LU31	Erba muraiola, erba murata	Aerial part	Decoction	(Flu) and sore throat	1 (PI)	No	Al	Wa, ru
		Aerial part	Infusion	For allergies	2(CH)	No	Sp	
Urtica dioica L. subsp. *dioica* MEB7	Ortica, 'rdica	Aerial part	Decoction (i.u.)	Spots on the skin	3 (PI)	No	Al	Unc,ru
		Aerial part	Decoction	Wash for itching	4 (VG)	No		
		Leaves	Cooked leaves are eaten	Anaemia	3 (CA)	No		
		Aerial part	Fresh crushed	On swellings, rheumatic pains, paralysis	3 (PE)<br>2 (CH)	No		
		Aerial part	Crushed in a mortar. Poultice (e.u.)	Sprains, bruises	4 (PI)	No		

		Rhizome (3 roots)	Decoction with 3 roots of mallow and 3 roots of *T. farfara*	Gastric pains	2 (AG)	Yes		
		A piece of stem with leaves	In a preparation (“uèvr’ “) with a nettle stem, one of ivy, a nail and the crest of a cock than never crows, gilded powder of a scratched bell, and a plant growing in wells similar to chicory	The ingredients were sewn into a small cloth bag of cloth and placed under children’s pillows to protect them from witches (magic-religious use)	1 (PI)	No		
**Valerianaceae**								
Valeriana officinalis L. MEA10	Vallariena	Root or leaves	Decoction (i.u.)	Hypertension	1 (PE)	Yes	Sp-Su	Da, cl
**Vitaceae**								
Vitis vinifera L. LU32	Vite	Vinegar	Compresses with water from the feet to the knees and on the forehead	Fever	3 (PI)	Yes (rare)	Al	Cu
		Red wine or must	Cooked until thick (i.u.)	Very strong astringent in case of diarrhoea and dysentery	4 (CH)	No	Al	
		Wine	External use	Wounds	1 (CP)	No	Al	
		Red wine	Hot wine with sugar (i.u.)	Bronchitis	4 (PG)	No	Al	
		Red wine	Hot wine with wood cinders (e.u.); then used for fumigations and footbaths	Bronchitis, cold	4 (PG)	No	Al	
		Red wine	Hot poultice with salt, covered with a wool cloth soaked in wine	Rheumatic pains	2 (AG)	No	Al	
		Vinegar	Compresses, mouthwashes	Fever (compresses), toothache (mouthwashes)	2 (AG)	No	Al	
		Vinegar	Compresses with hot water and salt	Dislocations	1 (AG)	No	Al	
		Unripe fruits	Juice	Drunk as astringent in case of severe diarrhoeas	4 (PG)	No	Su	

Abbreviations: **Localities**: AG = Agnone; BA = Baranello; CA = Castiglione di Carovilli; CH = Chiauci; CP = Capracotta; PE = Pescolanciano; PG = Poggio Sannita; PI = Pietrabbondante; PS = Pescopennataro; RC = Riserva di Collemeluccio; VG = Vastogirardi **Way of use**: i.u. = internal use e.u. = external use **Period of**

gathering Sp= spring; Su= summer; Fa= fall; Wi= winter; Al=always **Habitat** Cle = Clearings; Coh = near country houses; Cu= cultivated areas; Da = damp areas; Di = ditches; Dra = dry areas; Fi = fields; Kigd = kitchen gardens; He = hedges; Me = meadows; Mopa = mountain pastures; Pa = pastures; Ro = roadsides; Rock = rocky slopes; Ru = ruins; Shpl = shady places; Tewo = thermophilous woods; Unc = uncultivated areas; Vi = vineyards; Wa = walls; We = wells; Wo = woods.
